# Different Classes of Antidepressants Inhibit the Rat α7 Nicotinic Acetylcholine Receptor by Interacting within the Ion Channel: A Functional and Structural Study

**DOI:** 10.3390/molecules26040998

**Published:** 2021-02-13

**Authors:** Yorley Duarte, Maximiliano Rojas, Jonathan Canan, Edwin G. Pérez, Fernando González-Nilo, Jesús García-Colunga

**Affiliations:** 1 Center for Bioinformatics and Integrative Biology, Facultad de Ciencias de la Vida, Universidad Andrés Bello, Av. República 330, Santiago 8370146, Chile; yorley.duarte@unab.cl (Y.D.); maximiliano.rojas@unab.cl (M.R.); jonathan.canan@unab.cl (J.C.); fernando.gonzalez@unab.cl (F.G.-N.); 2 Interdisciplinary Centre for Neuroscience of Valparaíso, Facultad de Ciencias, Universidad de Valparaíso, Valparaíso 2381850, Chile; 3 Department of Organic Chemistry, Faculty of Chemistry and Pharmacy, Pontificia Universidad Católica de Chile, Santiago 7820436, Chile; eperezh@uc.cl; 4 Departamento de Neurobiología Celular y Molecular, Instituto de Neurobiología, Universidad Nacional Autónoma de México, Campus Juriquilla, Boulevard Juriquilla 3001, Juriquilla, Querétaro 76230, Mexico

**Keywords:** α7 nicotinic acetylcholine receptors, biological activity, hippocampus, antidepressants, in silico studies, allosteric modulators

## Abstract

Several antidepressants inhibit nicotinic acetylcholine receptors (nAChRs) in a non-competitive and voltage-dependent fashion. Here, we asked whether antidepressants with a different structure and pharmacological profile modulate the rat α7 nAChR through a similar mechanism by interacting within the ion-channel. We applied electrophysiological (recording of the ion current elicited by choline, I_Ch_, which activates α7 nAChRs from rat CA1 hippocampal interneurons) and in silico approaches (homology modeling of the rat α7 nAChR, molecular docking, molecular dynamics simulations, and binding free energy calculations). The antidepressants inhibited I_Ch_ with the order: norfluoxetine ~ mirtazapine ~ imipramine < bupropion ~ fluoxetine ~ venlafaxine ~ escitalopram. The constructed homology model of the rat α7 nAChR resulted in the extracellular vestibule and the channel pore is highly negatively charged, which facilitates the permeation of cations and the entrance of the protonated form of antidepressants. Molecular docking and molecular dynamics simulations were carried out within the ion−channel of the α7 nAChR, revealing that the antidepressants adopt poses along the receptor channel, with slightly different binding-free energy values. Furthermore, the inhibition of I_Ch_ and free energy values for each antidepressant-receptor complex were highly correlated. Thus, the α7 nAChR is negatively modulated by a variety of antidepressants interacting in the ion−channel.

## 1. Introduction

Cholinergic pathway dysfunctions have been associated with pathologies, such as Alzheimer’s disease, addiction, depressive disorders, and schizophrenia, among others [1,2,3,4]. With regard to depressive disorders, hyperactivation of the cholinergic pathway by nicotinic acetylcholine receptor (nAChR) agonists or acetylcholinesterase inhibitors brings forward depression in patients, even in adolescents [5,6,7,8].

nAChRs are pentameric proteins made up of the combination of α2–α10 and β2–β4 subunits, or forming homomeric receptors with a single subunit. nAChRs are non-selective cation channels permeable to Na^+^, K^+^, and Ca^2+^. The α4β2 and α7 nAChRs are the most abundant subtypes in the brain [9]. Although no model is available for the complete rat α7 nAChR, the X-ray structure of the human α4β2 nAChR has been obtained recently, as well as homology models developed for the human α7 nAChR using protein structures with high sequence identity with this receptor as templates [10,11].

nAChRs are regulated by a wide variety of substances, including antidepressants. In this regard, a common effect of several antidepressants with different pharmacological profiles (including tricyclic antidepressants, serotonin-specific reuptake inhibitors, and atypical antidepressants) is a non-competitive and voltage-dependent inhibition of nAChRs, consistent with molecular simulations, where the antidepressant-nAChR interaction takes place within the ion channel (i.e., the domain formed by the M2 transmembrane segment of each subunit) [12,13,14,15,16,17,18]. Furthermore, metabolites of some antidepressants, such as norfluoxetine (the main metabolite of fluoxetine), (*R*,*S*)-dehydronorketamine, and (*R*,*S*)-norketamine (ketamine metabolites) inhibit muscle and/or neuronal nAChRs [16,19].

It is well documented that the α7 nAChR is a target of multiple substances that include selective antagonists (methyllycaconitine), allosteric modulators, and anti-depressants, thereby, regulating depressive-like behavior in different murine models [20,21,22,23]. Furthermore, it is known that the α7 nAChR is highly expressed in the hippocampus, which is associated with depression, and that cholinergic signaling is increased during this condition [6,23]. It is well established over a long period of time that the α7 nAChR is a target of fluoxetine, which inhibits the functioning of this receptor [24]. Additionally, imipramine, bupropion, and mirtazapine also inhibit rat and/or human α7 nAChRs [12,15,17], restoring the cholinergic signaling, which is in agreement with the cholinergic hypothesis of depression [18]. Although several classes of antidepressants have been studied with in silico methods on different nAChR subtypes to support the existing experimental results [25], currently none of the antidepressants included here have been previously studied on the rat α7 nAChR with both functional and high-resolution structural approaches.

**Figure 1:**
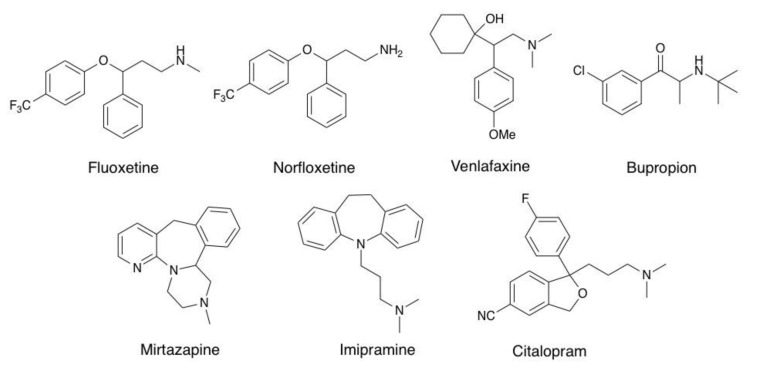
Chemical structures of antidepressants that modulate the α7 nicotinic receptor.

Thus, the aims of this work were (a) to study functional inhibitory effects of several antidepressants with different chemical structures and pharmacological profiles (Figure 1) on rat hippocampal α7 nAChRs, (b) to build a 3D structural model of the rat α7 nAChR by homology modeling, and (c) to correlate both functional (electrophysiological) and in silico approaches (molecular docking and molecular dynamics (MD) simulations and binding-free energy calculations) for the antidepressant-receptor interaction. This study acquires relevance in the sense that it contributes to the understanding of the structure-function relationships of nAChRs and the participation of these receptors, particularly the α7 nAChR, in the clinical treatment of major depression.

## 2. Results

### 2.1. Effects of Antidepressants on the Rat α7 nAChR

**Figure 2:**
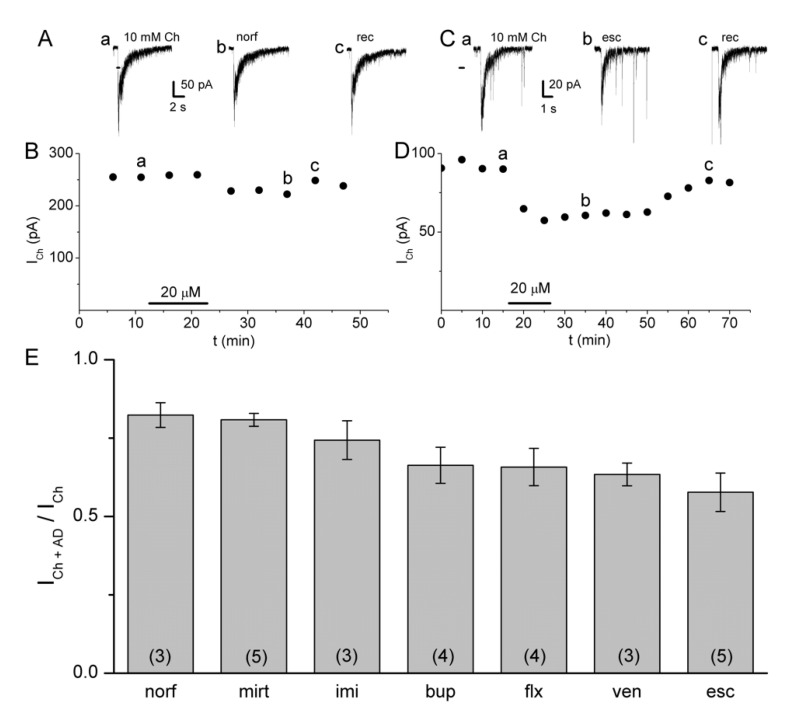
Samples of the current elicited by choline (I_Ch_) recorded in interneurons from the *stratum radiatum* rat hippocampal CA1 region and inhibited by 20 μM norfluoxetine (**A**,**B**) and 20 μM escitalopram (**C**,**D**) as a function of time. Data labeled as **a**, **b**, and **c** in (**B**,**D**) correspond to upper records in (**A**,**C**): control (**a**), maximal inhibited (**b**), and recovered (**c**) I_Ch_. (**E**) The height of the columns corresponds to the ratio I_Ch+AD_/I_Ch_ for each antidepressant at 20 μM. I_Ch+AD_ corresponds to the I_Ch_ in the presence of the corresponding antidepressant. From left to right: norf, norfluoxetine. mirt, mirtazapine. imi, imipramine. bup, bupropion. flx, fluoxetine. ven, venlafaxine. esc, escitalopram. Data correspond to the mean ± standard error. The number of independent experiments is in parenthesis. Comparison of the experimental data with their own control means was performed by paired Student’s *t*-test. *p* < 0.05 was considered statistically significant for all the antidepressants. The nonparametric Kruskal–Wallis test resulted in significant differences between different groups. The Mann Whitney Wilcoxon *Post hoc* analysis revealed no significant differences between pairs of groups.

It is known that several antidepressants modulate the functioning of nAChRs by a non-competitive inhibitory mechanism [12,15,16,17]. The electrical activity of native α7 nAChRs was recorded by applying local puffs of choline (Ch, a selective α7 nAChR agonist [26]) onto hippocampal interneurons. The resulting inward ion current elicited by Ch (I_Ch_) decayed even in the presence of the agonist due to receptor desensitization (Figure 2A,C). Note that noise considerably increases in the presence of the agonist due to the opening of receptors [27]. In some cases, fast downward responses were present, corresponding to spontaneous postsynaptic currents (Figure 2C). As control experiments, Ch-puffs were applied at 5-min intervals. The I_Ch_ amplitude remained constant for up to 60 min (*n* = 3, data not shown), indicating recovery of α7 nAChRs from desensitization in not more than 5 min. Furthermore, the I_Ch_ in *stratum radiatum* interneurons was completely inhibited by the selective antagonists methyllycaconitine and α-bungarotoxin, confirming the involvement of α7 nAChRs [17].

The antidepressants with different pharmacological profiles tested here were the selective serotonin reuptake inhibitors norfluoxetine, fluoxetine, and escitalopram, the tricyclic antidepressant imipramine, the α2 adrenergic receptor antagonist mirtazapine, the noradrenaline-dopamine reuptake inhibitor bupropion, and the serotonin-noradrenaline reuptake inhibitor venlafaxine [28]. This work complements and extends our previous studies regarding the interaction of antidepressants with the α7 nAChR [12,17,29]. Furthermore, none of the seven antidepressants included here have been previously studied on the rat α7 nAChR with both functional and high-resolution structural approaches. According to the IC_50_ values, or with the antidepressant concentration in which the ratio value (the I_Ch_ in the presence of an antidepressant to the control one: I_Ch+AD_/I_Ch_) was close to 0.5 μM, we observed antidepressants that exhibited low inhibitory potency to the rat α7 nAChR. Thus, this ratio for norfluoxetine and bupropion was 0.48 ± 0.03 and 0.52 ± 0.04, respectively, at 50 μM, for mirtazapine 0.56 ± 0.05 at 40 μM, whereas the IC_50_ for imipramine was 44.2 ± 8.5 μM [12]. Additionally, the antidepressants that displayed higher inhibitory potency for the receptor were fluoxetine and venlafaxine, with a ratio close to 0.5 at 25 μM, and escitalopram with an IC_50_ of 28.9 ± 5.1 μM, with similar inhibitory potency of fluoxetine for human α7 nAChR [24]. In this regard, we decided to perform a set of experiments by applying 20 μM of each antidepressant, a brain concentration reached after treatment of humans with fluoxetine [30,31], and rodents with (±)-citalopram or imipramine [32,33].

Thus, to explore the effects of antidepressants, Ch-puffs were initially applied at 5-min intervals to obtain the control I_Ch_. Thereafter, 20 µM of an antidepressant was added to the bath solution for ~10 min, resulting in a decrease of the I_Ch_ amplitude with respect to the control value. A sample of these inhibitory actions on I_Ch_ is illustrated for norfluoxetine and escitalopram. Under these experimental conditions, the inhibitory effect continued after washing out the antidepressant and the I_Ch_ recovered its control level in ~25 min (Figure 2A–D) due to slow removal of the drug from the brain tissue, similarly as in previous works [15,17]. The actions of the antidepressant were expressed as I_Ch+AD_/I_Ch_ and summarized in Figure 2E. At this concentration, all tested antidepressants showed inhibition of the α7 nAChR, with a sequence, from minimal to maximal inhibition: norfluoxetine ~ mirtazapine ~ imipramine < bupropion ~ fluoxetine ~ venlafaxine ~ escitalopram. Thus, norfluoxetine inhibited the control I_Ch_ by 17.7%, whereas escitalopram inhibited it by 42.3%. When the data were statistically analyzed, the nonparametric Kruskal–Wallis test showed that significant differences were detected between different groups of antidepressants (*H* (6) = 14.002, *p* = 0.029). However, Mann Whitney Wilcoxon *Post hoc* analysis revealed no significant differences between pairs of antidepressant groups, but differences close to the limit were obtained between mirtazapine and escitalopram (*p* = 0.063), and between norfluoxetine and escitalopram (*p* = 0.068).

### 2.2. Homology Modeling of the Rat α7 nAChR

We developed a homology model structure for the rat α7 nAChR to study the main interactions of the selected antidepressants. The available structure of the human α4β2 nAChR (PDB ID: 5KXI) was used as a template to build the pentameric rat α7 nAChR by homology modeling using Prime in Schrödinger Suite 2019-2 [10,34]. The identity between the two sequences is 48.47% and 66% coverage. These values are appropriate because, with the 5KIXI structure, there is greater identity in the pore area, which is particularly interesting for binding antidepressants. All these features make the α4β2 nAChR an ideal template for performing the homology modeling from their sequence alignment.

Since no structural information is available for the intracellular domain of the receptor, amino acid residues from 354 to 461 were deleted, directly linking residues 353 and 462. It was also considered that omission of these unmodelled residues does not affect functional properties of the human α4β2 nAChR [10]. Although it is not discarded, the possibility that antidepressants interact at the orthosteric site (for imipramine and escitalopram at the (α9)_2_(α10)_3_ nAChR) [12,13], the present study focuses on the interaction between antidepressants and the α7 nAChR, which takes place within the ion channel, lined by the M2 transmembrane segment of each subunit, which is a common binding site of the most studied antidepressant [12,15,17]. Then, the homology model of a single α7 nAChR subunit with the lowest potential energy was chosen to build the homomeric pentamer.

**Figure 3:**
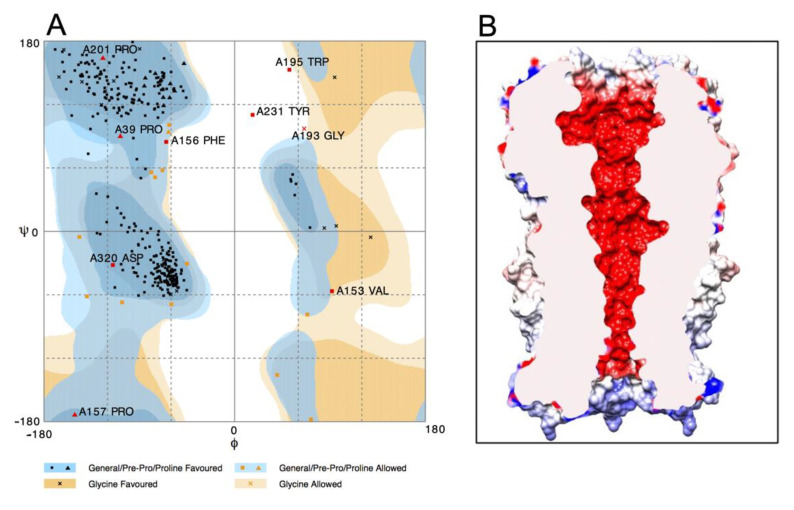
(**A**) Ramachandran plot of the rat α7 nAChR model generated by the RAMPAGE server. (**B**) Electrostatic potential of the α7 nAChR. The electronegative region is illustrated in red whereas electropositive is illustrated in blue (values scale is shown in ±10 kT/e).

The quality of the modeled structure was evaluated to determine its reliability. We used the RAMPAGE program (Ramachandran plots using the Richardsons’ data) to verify the stability of the stereochemical parameters [35]. The Ramachandran plot shows a correct conformation of the secondary structure for the α7 nAChR, showing that around 93.7% of the amino acids fall within the most favored regions, 4.6% of the residues fall in allowed regions, and only 2.6% fall in the outlier region, indicating that the homology model is of good quality (Figure 3A) [10,36].

The α7 nAChR model was equilibrated using 100 ns MD simulations, which were performed using the Desmond module of Schrödinger. The RMSD (Root Mean Square Deviation) serves as a measure to determine the stability of the α7 nAChR structure based on its deviation from the initial structure. This calculation showed that the protein remained stable for the last 80 ns of the Molecular Dynamic trajectory (Supplementary Materials Figure S1).

**Figure 4:**
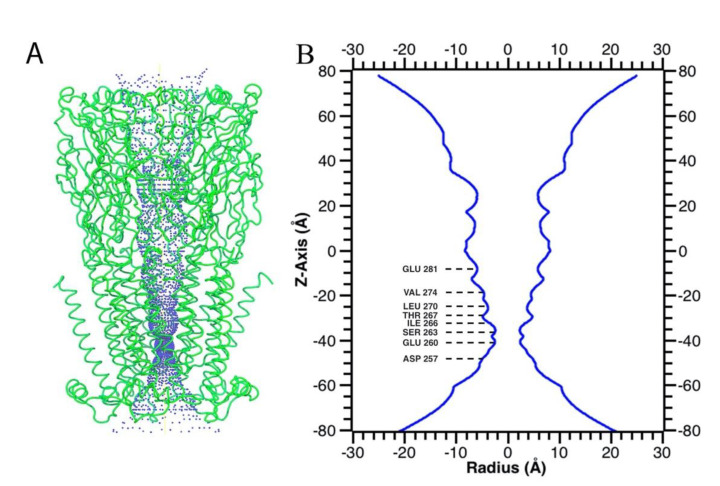
The narrowest central region of the ion channel illustrates residues directed toward the channel lumen. (**A**) α7 nAChR receptor pore domain highlighting the pore size in blue. (**B**) Pore radius of the receptor. Lines indicate the residues facing the ion channel.

The electrostatic potential of the α7 nAChR model was evaluated to probe ion selectivity mechanisms. The region of the extracellular vestibule and the ion channel are highly electronegative, which facilitates increased concentration and passage of permeable cations (Na^+^, K^+^, and Ca^2+^) and the entrance of other electropositive molecules, such as antidepressants, in their protonated forms, whereas the intracellular region is electropositive (Figure 3B). The narrowest part of the ion channel is located at the level of amino acids E260 and S263, with a diameter of ~5 Å (Figure 4A,B), which is greater than the diameter of the partially hydrated, permeable cations.

### 2.3. Molecular Docking

The predicted model binding site for antidepressants needs to be identified for docking studies. Due to the unavailability of an α7 nAChR crystal structure, the homology model binding site was sought along the ion channel from the extracellular region (L278, 16′ ring) to the cytoplasmic side (S263, 2′ ring) (Figure 4A,B). A docking study was performed to elucidate the binding mode of each antidepressant in the α7 nAChR, and the compounds were docked using ‘extra precision’ glide docking (Glide XP), which docks compounds flexibly. Docking results for each antidepressant into the α7 nAChR homology model provided several configurations that were scored by Glide to determine favorable binding modes. Comparison of the different docking poses of antidepressants revealed similar binding interactions within the ion channel, located mainly between residues S263 and V274 2′ and 13′ rings (Figure 4). The key interactions were dominated by S263, I266, T267, T273, and Val274. These interactions are in agreement with the earlier observed binding modes (Supplementary Table S1) [12].

### 2.4. Molecular Dynamics Simulation of Top Binding Poses

**Figure 5:**
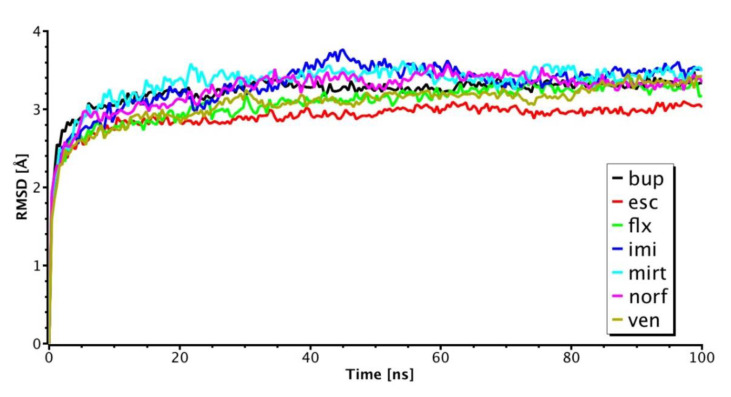
The RMSD plot for each antidepressant-receptor complex. bup, bupropion. esc, citalopram. flx, fluoxetine. imi, imipramine. mirt, mirtazapine. norf, norfluoxetine. ven, venlafaxine.

**Figure 6:**
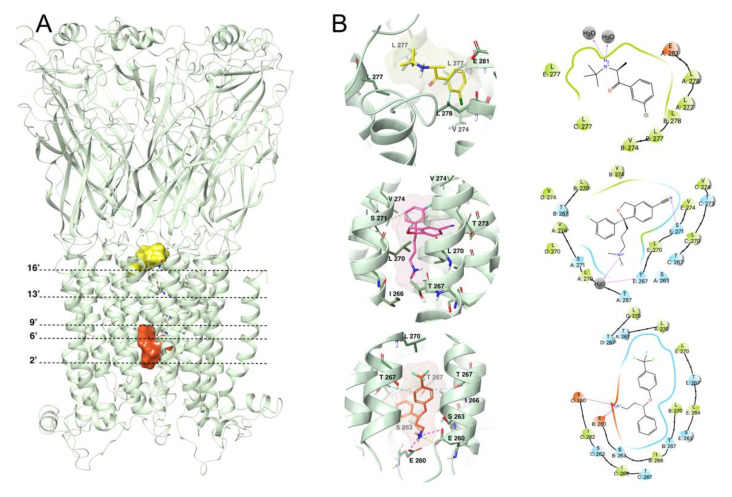
(**A**) Representation of the general location of antidepressants in the ion channel. The position of the representative rings is indicated by dotted lines. (**B**) Images corresponding to the last conformation of bupropion (yellow), escitalopram (pink), and norfluoxetine (orange) in the ion channel of the rat α7 nAChR homology model binding pocket after a 100 ns Molecular Dynamics simulation. Hydrophobic interactions, H-bonds, and ionic interactions are shown in cyan, yellow, and fuchsia, respectively.

To investigate the dynamics of antidepressant binding, 100 ns MD simulations were performed for each antidepressant-α7 nAChR complex. The starting conformation of each complex was taken from the flexible molecular docking poses. The fluctuations of antidepressants and the receptor during the MD simulations were evaluated by root-mean-square deviation (RMSD) against an initial structure. For antidepressant-α7 nAChR complexes with escitalopram, venlafaxine, and fluoxetine, the fluctuation of the RMSD values was relatively small with values less than 3.0 Å, showing stability during the simulations (Supplementary Figure S2) and Root-mean-square fluctuations (RMSF) for each antidepressant. The RMSD plots for the antidepressant-receptor complexes mentioned above are shown in Figure 5. For the other three systems, i.e., norfluoxetine, mirtazapine, and imipramine, the RMSDs were in the 3.2–3.5 Å range, suggesting that the initial binding mode of these antidepressants is not maintained throughout the MD simulations, generating significant conformational changes in the receptor. We illustrated the interaction as well as the amino acid residues establishing contacts between the weakest (norfluoxetine), the intermediate (bupropion), and the strongest (escitalopram) inhibitor and the α7 nAChR for the last conformation in the ion channel after 100 ns MD. Most of the poses for all the antidepressants tested were located between S263 and V274 (2′and 13′ rings) except for bupropion (Figure 6A,B). The antidepressant-receptor complex interactions were mainly through van der Waals contacts, and, in some cases, through hydrogen bonds (Figure 6B, Table 1).

### 2.5. MM-GBSA Calculations

**Figure 7:**
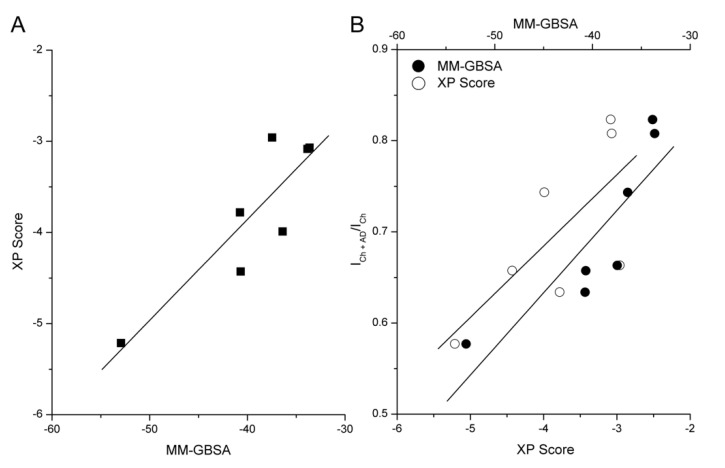
Correlation between molecular docking XP score and MM-GBSA values (**A**). Correlations between the biological activity and both the docking XP score and MM-GBSA (**B**). Data were fitted using a linear regression.

The binding-free energy of each antidepressant was calculated during the 100 ns of MD simulation to get a better estimate of the binding strengths and relative potencies against α7 nAChR. In general, the calculated binding energies obtained with MM-GBSA for the antidepressants tested went from −33.6 to −52.9 kcal/mol (Table 1), with escitalopram having the lowest energy value and norfluoxetine having the highest value. Furthermore, the extra precision XP score obtained for each antidepressant-receptor docking were correlated with the free energy values obtained by Molecular Mechanics Generalized Born Surface Area (MM-GBSA) (Figure 7A), showing a Pearson correlation coefficient of 0.88 (associated *p*-value of 0.0093). On the other hand, the correlations of biological activity (the inhibition of the ion current mediated by α7 nAChR and evaluated by the ratio I_Ch+AD_/I_Ch_) with in silico results, XP scores, and the free energy values (Figure 7B) resulted in Pearson correlation coefficients of 0.71 (*p*-value 0.0764) and 0.86 (*p*-value 0.0125), respectively.

## 3. Discussion

In this work, the inhibitory activity of antidepressants with a different structure and pharmacological profile was studied on rat hippocampal α7 nAChRs, and then was correlated with the antidepressant-receptor interactions found through in silico studies in a homology model of the rat α7 nAChR.

Several antidepressants inhibit nAChRs in a non-competitive and voltage-dependent way, suggesting a common mode of action, binding affinity, and interacting site within the ion channel of these receptors [12,14,15,16,17,18]. Particularly, the substances studied here (bupropion, escitalopram, fluoxetine, imipramine, mirtazapine, norfluoxetine, and venlafaxine), at a concentration of 20 μM, inhibited α7 nAChRs from 17.7% to 42.3% of the control response. We consider that these findings may have clinical relevance because this is a brain concentration reached after treatment of depressed humans with fluoxetine [30,31], corresponding to brain concentration after treatment of rodents with (±)-citalopram or imipramine [32,33]. An antidepressant concentration that, at least partially, decreases the activity of hippocampal α7 nAChRs.

Accordingly, it is known that the α7 nAChR is widely expressed in the hippocampus, known as a brain region associated with depression. Cholinergic signaling is increased among those with this disorder [6,23]. In this sense, the inhibition of hippocampal α7 nAChRs by the antidepressants studied here may recover the cholinergic signaling, which is in accordance with the cholinergic hypothesis of depression [18]. Likewise, the non-selective, α4β2-selective, and particularly α7-selective antagonists, e.g., mecamylamine, dihydro-β-erythroidine, and methyllycaconitine, respectively, have antidepressant-like effects [37]. In this regard, there is increasing evidence that other antidepressants and substances with antidepressant-like effects also inhibit the α7 nAChR: duloxetine, ketamine, methyllycaconitine, or ligands for peroxisome proliferator-activated receptors type-α [19,23,38,39].

The homology model of the rat α7 nAChR built here was generated using the structure of the human α4β2 nAChR [10] as a template, obtaining similar physical and functional properties, in terms of electronegativity in the extracellular vestibule and ion channel, and the narrowest diameter of the ion channel. Recently, a homology model of the human α7 nAChR was built, by using the mouse 5-HT_3A_ receptor structure as a template, for studying interactions with positive allosteric modulators, in which the residue sequence identity between these receptors was 28.7% [40], compared with 48.47% of identity obtained in the actual study.

Since the I_Ch_ inhibition by antidepressants is correlated with both the molecular docking XP score and the binding free energy calculated during the MD simulation (Figure 7), we found that those antidepressants that showed less inhibitory potency interact with sites located closer to the cytoplasmic side between rings 2′ and 9′, displaying a less favorable docking XP score and binding-free energy values norfluoxetine, mirtazapine, and imipramine (Figure 6). Conversely, those antidepressants that showed high inhibitory potency also interact with sites located closer to the extracellular mouth of the ion channel between rings 9′ and 16′, exhibiting more negative values of the docking XP score and binding free energy: bupropion, fluoxetine, venlafaxine, and escitalopram (Table 1, Supplementary Tables S2 and S3). These energy values reflect stronger binding of these antidepressants to the receptor, especially escitalopram, in agreement with the experimental results.

We compared the different regions where antidepressants interact in the ion channel. Thus, in the human α7 nAChR, the biding sites for imipramine are in rings 2′, 9′, and 13′, compared with a more restricted region in the rat α7 nAChR (Figure 6, Supplementary Table S2 [12]). Bupropion biding sites are very similar in both human and rat α7 nAChRs (Supplementary Table S2 [17]). In the case of fluoxetine, the binding sites have a more restricted region in human α7, α3β4, and α4β2 nAChRs [41] compared with the rat α7 nAChR (Supplementary Table S3). Note that there are some subtle differences between the interactions of the above antidepressants with the rat and human α7 nAChRs, even though the M2 segments are identical in both species. In addition, escitalopram interacts in the same region in the human α3β4 [13] and in the rat α7 nAChRs, between rings 6′ and 13′ (see Supplementary Table S3) whereas the ketamine binding site is located at ring 9′ in the human α7 nAChR [42].

Analysis of the main contacts that the antidepressants generated over the MD simulation trajectory of 100 ns revealed that, for norfluoxetine E260 and I266 of two chains in the protein, occupancy of more than 50% was presented throughout the trajectory, mainly stabilized by hydrogen bonds between the acid group of glutamate and the protonated amine group of norfluoxetine, as well as hydrophobic interactions with aromatic rings. On the other hand, T267, L270, S271, and V274 of the five chains of the α7 nAChR played an important role in the protein-escitalopram interactions through hydrophobic contacts and water-mediated hydrogen bonds over much of the trajectory (Figure 6). For the receptor-bupropion complex, L277 and L278 of three of the five chains, presented hydrophobic interactions with an occupancy of less than 50% during the MD trajectory (Figure 6). Furthermore, for venlafaxine and mirtazapine in complex with the receptor, the cation–π or hydrogen bond interactions of E260 and S263 with the protonated amine of each antidepressant was the most prevalent interaction with an occupancy of more than 50%. However, in the receptor-venlafaxine complex, the hydrophobic interactions with I266 and L270 of four chains also had high prevalence. Finally, imipramine and fluoxetine in a complex with the α7 nAChR showed similar binding interactions with L270 and T267. These small differences in protein-antidepressant interactions slightly altered the binding-free energy values (MM-GBSA) of each complex.

These findings are also consistent with values of the electric distance (fraction of the electrical field sensed by the compound at its binding site in the ion channel), which ranged from 0.10 to 0.40 for different antidepressants [12,13,16,43]. It is interesting to note that, in addition to antidepressants, other substances interact within the ion channel of nAChRs in similar regions, including tetracaine, carbamazepine, barbiturates, and anesthetics, such as phencyclidine and ketamine [12,13,14,15,17,29,44]. All this indicates that the M2 domain lining the ion channel of nAChRs is very reactive, having specific binding sites for a variety of non-competitive allosteric modulators. That is the case for analogs of methyllycaconitine, which competitively inhibit the rat α7 nAChR, whereas they inhibit the rat α4β2 nAChR in a non-competitive and voltage-dependent manner by lodging between rings 6′ and 13′ [45].

Considering that a more negative binding energy corresponds to a stronger biological activity (inhibitory potency) [46], we obtained a very good correlation between the pharmacological activity of the antidepressants, measured electro-physiologically, and the XP score for each antidepressant-receptor docking and the free energy. Furthermore, in accordance with the free energy values (Table 1), the interaction of escitalopram and venlafaxine with the α7 nAChR ion channel is more favorable than the interaction of mirtazapine, which is consistent with the most stabilized docking in the ion channel, whereas the most unstable dockings are those with mirtazapine and imipramine. Additionally, functional results indicated that escitalopram and venlafaxine were the most potent inhibitors of the α7 nAChR. Fluoxetine and norfluoxetine are the most potent inhibitors for other nAChRs [14,16].

According to both pharmacological activity and molecular simulations, we conclude that a variety of antidepressants with a different chemical structure and pharmacological profiles similarly inhibit the rat α7 nAChR by interacting within the ion-channel with slight differences in binding-free energy. These results help us understand the interaction between antidepressants and nAChRs and their therapeutic and/or adverse effects of these compounds using nAChRs as targets.

## 4. Materials and Methods

### 4.1. Electrical Recordings in Hippocampal Slices

All experimental procedures were carried out in accordance with the National Institute of Health Guide for Care and Use of Laboratory Animals and were approved by the Institutional Animal Care Committee of the Universidad Nacional Autónoma de México, with an effort to minimize the number of animals used and their suffering.

The experiments were performed as previously described [15]. Sprague Dawley rats on postnatal days 13–16 were deeply anesthetized with isoflurane and then decapitated. Their brains were removed and placed into an ice-cold (4 °C) solution containing (in mM): 250 sucrose, 2.5 KCl, 1.2 NaH_2_PO_4_, 5 MgCl_2_, 0.5 CaCl_2_, 26 NaHCO_3_, and 10 glucose (pH 7.4). Coronal slices (350-µm thick) containing the hippocampal CA1 area were cut with a Vibratome Leica VT 1000S and submerged in artificial cerebrospinal fluid (ACSF) containing (in mM): 125 NaCl, 2.5 KCl, 1.23 NaH_2_PO_4_, 1 MgCl_2_, 2 CaCl_2_, 26 NaHCO_3_, and 10 glucose (pH 7.4). The slices were stabilized in this solution for at least 1 h before electrical recording. All solutions were continuously bubbled with 95% O_2_ and 5% CO_2_ at room temperature.

One slice was transferred into a chamber and super-fused with ACSF at a rate of ~2 mL/min. Interneurons were visualized using an infrared video-microscopy system (BX51WI, Olympus Instruments, Tokyo, Japan) endowed with an 80× water immersion objective. Whole-cell voltage-clamp recordings [47] were performed with a PC-ONE Patch/Whole Cell Clamp (Dagan Corporation, Minneapolis, MN, USA). The signal was passed through a 3-pole lowpass Bessel filter at 3 kHz and acquired at 10 kHz with a Digi-data 1440A A/D converter driven with pClamp 10 (Molecular Devices, Sunnyvale, CA, USA). Patch-clamp electrodes had a resistance of 3–7 MΩ when filled with the internal solution (in mM): 140 K-gluconate (or KCl), 10 2-[4-(2-hydroxyethyl)piperazin-1-yl]ethanesulfonic acid (HEPES), 2 MgCl_2_, 0.5 CaCl_2_, 10 ethylene glycol-bis(β-aminoethyl ether)-*N*,*N*,*N*′,*N*′-tetraacetic acid (EGTA), and 2 MgATP (pH 7.4). Similar results were obtained by indistinctly using each of these internal solutions. Recorded interneurons were in the *Striatum radiatum* hippocampal CA1 area and were maintained at a potential of −70 or −20 mV.

The procedure for exploring the effects of antidepressants was previously described [15,17]. Choline (Ch, 10 mM) puffs (2–5 psi, 500 ms) were applied onto interneurons through a glass micropipette placed ~10 µm from the recorded cell by using a pneumatic pico-pump (PV830, WPI, Sarasota, FL, USA). All drugs (norfluoxetine, fluoxetine, imipramine, bupropion, escitalopram, venlafaxine, mirtazapine, and choline) were obtained from Sigma-RBI. Thus, Ch-puffs were applied 5 min before, during, and after the desired antidepressant was added to the bath solution for ~10 min. The amplitude of currents elicited by Ch (I_Ch_) was measured as a function of recording time.

The I_Ch_ amplitude in the absence or presence of the antidepressant was measured with pClamp 10 software. Origin 7 (MicroCal Software, Northampton, MA, USA) was used to analyze, fit, and graph the results. Data are presented as a mean ± standard error. Comparison of the experimental data with their own control means was performed by paired Student’s *t*-test. The nonparametric Kruskal–Wallis test was performed to evaluate differences between groups. Then, the Mann Whitney Wilcoxon *Post hoc* test was used to examine significant differences between pairs of groups. In all cases, *p* < 0.05 was considered statistically significant.

### 4.2. Homology Modeling of the Rat α7 nAChR

For homology modeling of the rat α7 nAChR, we used the amino acid sequence of *Rattus norvegicus* α7 nAChR (uniprot code Q05941). The homology model of the α7 nAChR was built with the crystallographic data of the human α4β2 nAChR [PDB: 5KXI) [10]. Ten homology models were generated by prime, and the model with the lowest potential energy-OPLS3e [48,49,50] was chosen to continue studies of a protein-ligand interaction. An α7 monomer of the nAChR was modeled and used as a base to build the pentamer. The final model was visually inspected with verification of the secondary structure compared to the crystal, taking the coverage of the residues of interest as a reference with the crystal [48]. After building the model, its quality and most common structural problems were optimized using the Protein Preparation Wizard module [51]. The 3D model generated was structurally minimized and equilibrated using Desmond Schrödinger software [52] and further checked for stereochemical quality by Ramachandran plot analysis using the RAMPAGE server (Ramachandran plots using the Richardsons’ data) [35]. All procedures for searching sequence alignment, the template, and generation of the homology modeling were performed with the Prime suite in Schrödinger 2019-2 software LLC, New York, NY, USA [34].

### 4.3. Molecular Docking

The antidepressants were docked using the Glide [53] program in Schrödinger 2019-2 [51] using default settings. The OPLS3e force field was used for the docking protocol [48]. The antidepressant structures were drawn in 2D sketcher and prepared with the ligprep suite. Since the most common effect of antidepressants on nAChRs is voltage-dependent inhibition, indicating that these compounds interact within the ion channel of the receptor [15,16,54], the grid was fixed to contain the complete ion channel spanning the membrane. First, to look for the interacting site of the antidepressants, molecular docking was achieved with the standard-precision (SP) protocol. Then, the region with the greatest molecular clustering was selected as the active binding site. The grid dimension for testing the antidepressant docking was a 12 × 12 × 12 Å cube centered close to the narrowest part of the ion channel. Then, the molecular docking for each antidepressant was performed on the α7 nAChR using the extra-precision (XP) protocol. All these procedures were performed using Schrödinger 2019-2 software, LLC, New York, NY, USA [51].

### 4.4. Molecular Dynamics Simulations

We performed an independent MD simulation for each antidepressant-receptor complex using the Desmond program in Schrödinger 2019-2 [52]. Each antidepressant-receptor complex was inserted into a POPC (1-palmitoyl-2-oleoylphosphatidylcholine) membrane of approximately 140 × 140 Å and solvated using explicit TIP3P water models in an orthorhombic box with periodic boundary conditions. All complexes were neutralized with 0.15 mol/L of NaCl and parametrized with the OPLS3e force field [48,49,50]. Isothermal–isobaric ensemble NPT at standard conditions of T = 310.15 K and *p* = 1 atm were used. The complexes were subjected to the minimization protocol based on the steepest descent method, with the annealing steps of 2000 and 100 ps steps [55]. Each simulation was performed for a total of 100 ns, with recording intervals of 100 ps. Within the parameters of the Schrödinger software, the equilibration and relaxation protocol were selected for a membrane system in a molecular dynamics panel prior to the production of MD [56].

### 4.5. Free Energy (MM-GBSA) Calculations

MM-GBSA (Molecular Mechanics-Generalized Born Surface Area) was used to estimate the binding free energy of each antidepressant-receptor complex. For the MM-GBSA calculation, only 20 ns simulation were considered, which correspond to the frames with the lowest fluctuation during the dynamics (based on the RMSD and RMSF) [57]. For the surface area, the MM/GBSA approach was used as implemented in the Prime [34,58] module of Schrödinger 2019-2 using the default settings [51]. The MM-GBSA analysis was performed on three subsets of each system: the receptor alone, the antidepressant alone, and the complex (antidepressant-receptor). The total free energy was calculated, including all the molecular mechanics contributions (bond, angle, and dihedral energies, electrostatic and van der Waals energies). To calculate the MM-GBSA during the MD simulation, a Thermal MMGBSA script was used. This script takes in a Desmond MD trajectory, splits it into individual frame snapshots, and runs each one through MM-GBSA (after deleting waters and separating the ligand from the receptor).

## Figures and Tables

**Table 1:** Main interactions and binding-free energies of the antidepressants in the α7 nAChR.

Antidepressant	Contacts with Receptor Residues	ΔG (Kcal/mol)
norfluoxetine	*Van der Waals:* L270, I266 *Hydrogen bond:* E260	−33.8 ± 3.19
mirtazapine	*Van der Waals:* L270, S263, T267, I266 *Hydrogen bond:* E260	−33.6 ± 2.50
imipramine	*Van der Waals:* T267, I266, L270	−36.4 ± 3.03
bupropion	*Van der Waals:* L278, L277, V274	−37.5 ± 3.07
fluoxetine	*Van der Waals:* L270, S263, I266 *Hydrogen bond:* T267	−40.7 ± 2.56
venlafaxine	*Van der Waals:* L270, S263, T267, I266 *Hydrogen bond:* E260	−40.7 ± 3.38
escitalopram	*Van der Waals:* L270, T267, V274 *Hydrogen bond:* S271, T267	−52.9 ± 4.34

## Data Availability

The data presented in this study are available in article and Supplementary Materials here.

## Supplementary Materials

The Supplementary Materials are available online. Supplementary Figure S1. Last 100 ns of the molecular dynamics of the α7 nAChR backbone. Supplementary Table S1. Main interactions obtained from XP molecular docking and binding docking score values of the anti-depressant in the α7 nAChR. Supplementary Figure S2. RMSF plots for each residue of the α7 nAChR. Supplementary Table S2. Results for Biological Activity, MMGBSA, and molecular docking. Supplementary Table S3. Amino acid residues that contact antidepressants.

## Sample Availability

Samples of the compounds are not available from the authors.
