# Supplementary material for: Different Classes of Antidepressants Inhibit the Rat α7 Nicotinic Acetylcholine Receptor by Interacting within the Ion Channel: A Functional and Structural Study
Source: Molecules. 2021 Feb 13;26(4):998. doi: 10.3390/molecules26040998 (PMC7918632; doi:10.3390/molecules26040998)
Supplement: Supplementary file 1 [file molecules-26-00998-s001.pdf]

Supplementary Material

Different classes of antidepressants inhibit the rat α7 nicotinic acetylcholine receptor by interacting within the ion channel: a functional and structural study

Yorley Duarte, Maximiliano Rojas, Jonathan Canan, Edwin G. Pérez, Fernando González-Nilo and Jesús García-Colunga.

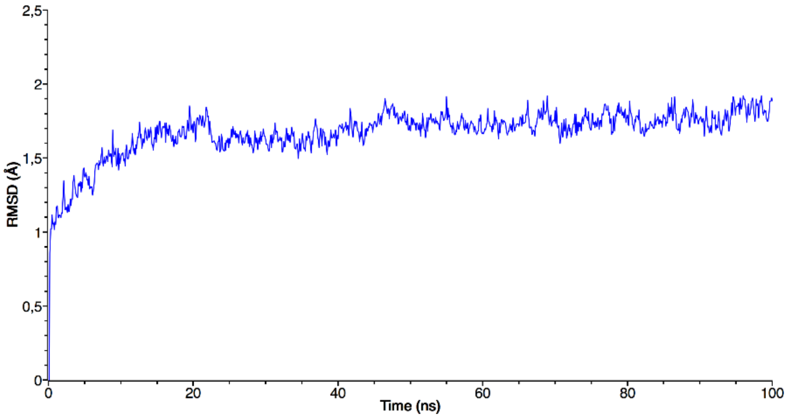

Supplementary Figure S1. Last 100 ns of the molecular dynamics of the α7 nAChR backbone. The RMSD values as a function of time.

Supplementary Table 1. Main interactions obtained from XP molecular docking and binding docking score values of the antidepressant in the α7 nAChR.

**Antidepressant**	**Contacts with receptor residues**	**Docking score (kcal/mol)**
norfloxetine	*Van der Waals:* L270, I266, T267 *Hydrogen bond:* S263	-3.1
mirtazapine	*Van der Waals:* L270, S263, T267, I266	-3.1
imipramine	*Van der Waals:* L270, T267, I266	-4.0
bupropion	*Van der Waals:* L277, V274 *Hydrogen bond:* T273	-3.0
fluoxetine	*Van der Waals:* L270, V274, I266, T267 *Hydrogen bond:* S263	-4.4
venlafaxine	*Van der Waals:* S263, I266 *Hydrogen bond:* E260, S263	-3.8
citalopram	*Van der Waals:* L270, T267, V274, I266, S271 *Hydrogen bond:* T273	-5.2

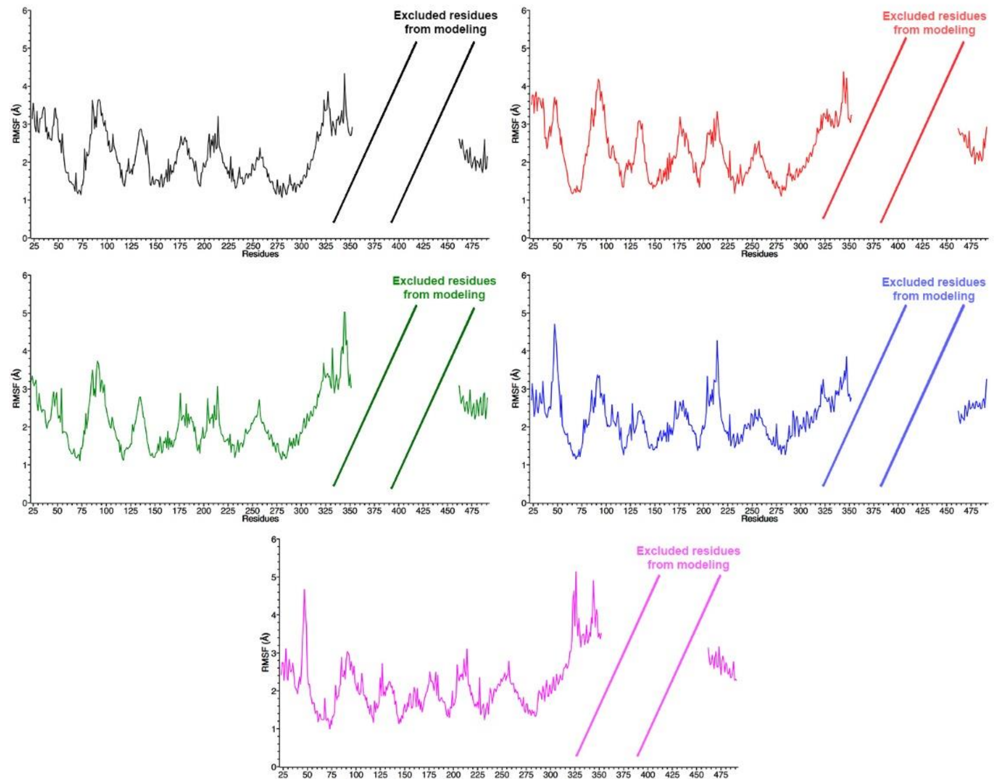

Supplementary Figure S2. RMSF plots for each residue of the α7 nAChR. Bupropion in black, citalopram in red, fluoxetine in green, imipramine in blue and norfluoxetine in pink.

Supplementary Table 2. Results for Biological Activity, MMGBSA and molecular docking.

**Antidepressant**	**Biological Activity ($I_{Ch + AD}$ / I_{Ch})**	**Free energy (MMGBSA) Kcal/mol**	**Docking XP score Kcal/mol**
norfloxetine	0.823 ± 0.039	-33.847	-3.085
mirtazapine	0.808 ± 0.021	-33.633	-3.07
imipramine	0.743 ± 0.062	-36.387	-3.99
fluoxetine	0.663 ± 0.058	-40.689	-4.429
venlafaxine	0.657 ± 0.059	-40.749	-3.781
bupropion	0.634 ± 0.036	-37.459	-2.96
escitalopram	0.577 ± 0.061	-52.940	-5.213

Supplementary Table 3. Amino acid residues that contact with antidepressants.

				2´				6´			9´				13´				17´			
rα7	E	K	I	S	L	G	I	T	V	L	L	S	L	T	V	F	M	L	L	V	A	E
				263				267			270				274				278			
norf				x			x	x			x											
mirt				x			x	x			x											
imi							x	x			x											
bup												x		x	x	x		x				
flx				x			x	x			x				x							
ven	x			x			x															
esc							x	x			x	x		x	x							
